# Piperazine Derivatives: A Privileged Scaffold in Modern Synthesis and Medicinal Chemistry

**DOI:** 10.1002/open.202500366

**Published:** 2026-01-19

**Authors:** Assel Ten, Raushan Koizhaiganova, Dilnaz Bissenbay, Bagila Tursynova, Zhanar Zhaxibayeva, Valentina Yu

**Affiliations:** ^1^ Laboratory of Synthetic and Natural Medicinal Compounds Chemistry A.B. Bekturov Institute of Chemical Sciences Almaty Kazakhstan; ^2^ Faculty of Chemistry Al‐Farabi Kazakh National University Almaty Kazakhstan; ^3^ Department of Chemistry Kazakh National Pedagogical University named after Abai Almaty Kazakhstan

**Keywords:** anticancer agents, antimicrobials, antipsychotics, heterocyclic synthesis, piperazine, privileged scaffold, structure–activity relationship

## Abstract

Interest in piperazine scaffolds continues to rise due to their broad relevance across anti‐infective, anticancer, and neuroactive research. This review examines reports published from 2014 to 2024 and organizes current developments by therapeutic class, structural modification strategy, and computational assessment. Substitution patterns involving aryl, heterocyclic, and hybrid groups show consistent effects on target affinity, selectivity, and pharmacokinetic properties. Several series demonstrate strong activity in early biological evaluation, supported by docking and pharmacodynamic trends that highlight recurring structural motifs. Synthetic approaches, including *N*‐functionalization, reductive routes, cross‐coupling, C—H activation, microwave‐assisted reactions, and flow‐based methods, provide diverse access to optimized derivatives. Combined interpretation of synthetic, biological, and computational results outlines reproducible structure–property relationships that guide piperazine‐focused design. Future progress is expected to arise from hybrid scaffold engineering, improved strategies for central nervous system delivery, and the integration of predictive machine‐learning methods into lead refinement.

## Graphical abstract

Piperazine‐based bioactive molecules represent a versatile class of compounds with broad therapeutic potential. Structural modification of the piperazine scaffold governs key structure–activity relationships, enabling antibacterial, antifungal, antitumor, neuroactive, and anti‐inflammatory activities. Diverse synthetic functionalization strategies support targeted substitution patterns that enhance potency, selectivity, and pharmacological performance.

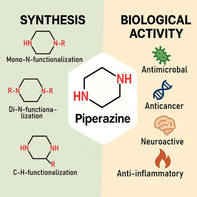

## 1 Introduction

The piperazine heterocycle is one of the most widely used and chemically versatile scaffolds in modern medicinal chemistry. Structurally, it comprises a saturated six‐membered ring with two nitrogen atoms at the 1 and 4 positions, conferring conformational flexibility and tunable basicity that are essential to its function [1, 2]. This dual‐nitrogen architecture enables the ring to adopt both chair and boat conformations, favoring molecular fit within enzyme active sites, receptor pockets, and transporter cavities. It also supports multiple noncovalent interactions, including hydrogen bonding, cation‐π contacts, and electrostatic interactions, which enhance ligand‐target affinity and broaden pharmacological potential [3, 4]. The secondary amine groups allow precise modulation of protonation states and charge distribution, properties that affect absorption, membrane permeability, and pharmacokinetic behavior. Due to this combination of structural simplicity and chemical adaptability, piperazine is widely regarded as a privileged scaffold in drug discovery [5]. Its nitrogen atoms act as versatile synthetic handles, enabling systematic functionalization to fine‐tune steric, electronic, and physicochemical properties such as aqueous solubility, lipophilicity, and pK_a_ [6, 7]. This synthetic tunability facilitates the optimization of absorption, distribution, metabolism, and excretion (ADME) profiles and supports high‐affinity binding across a range of biological targets. Piperazine cores are incorporated into drug design and lead‐optimization campaigns to enhance potency, selectivity, and bioavailability [5, 8, 9].

**FIGURE 1:**
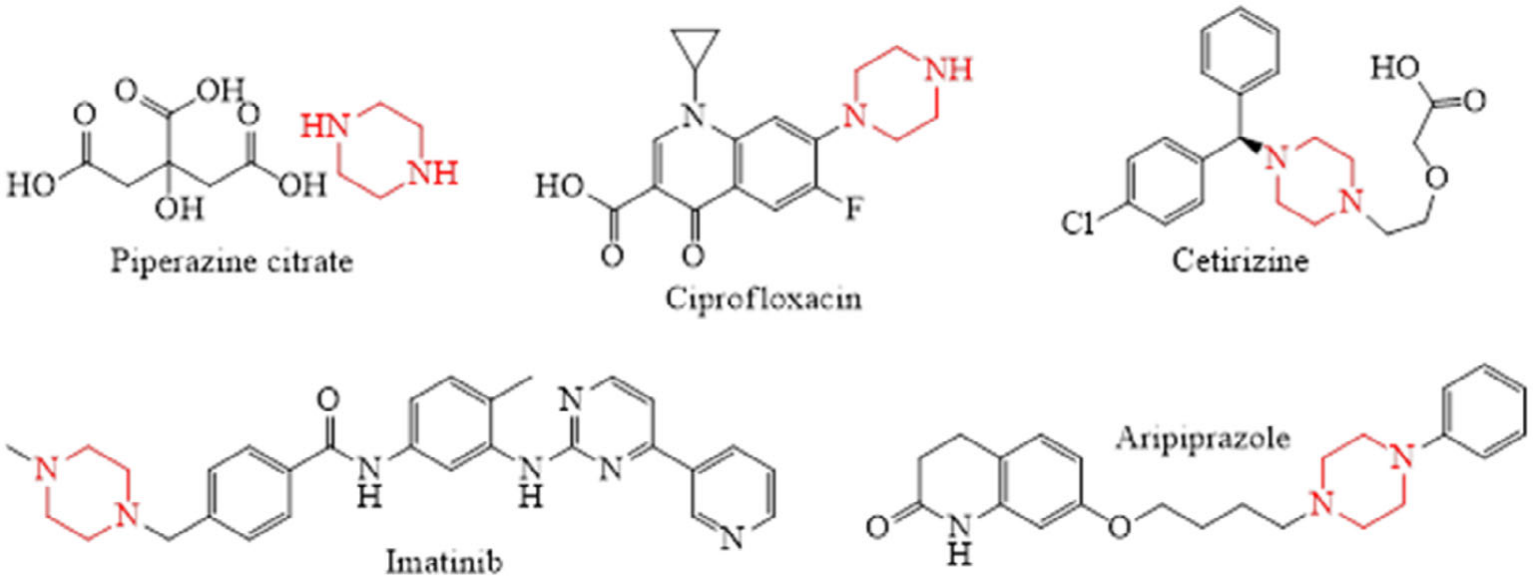
Representative piperazine‐containing drugs highlighting the structural versatility and pharmacological relevance of this scaffold.

The clinical relevance of this scaffold is shown by its presence in numerous marketed drugs across major therapeutic classes. For instance, the anticancer agent imatinib exploits a piperazine moiety to increase kinase selectivity [10, 11], while the atypical antipsychotic aripiprazole uses the heterocycle to adjust dopamine and serotonin receptor binding [12]. The widely prescribed fluoroquinolone antibiotic ciprofloxacin incorporates a piperazine ring to increase bacterial enzyme binding and membrane permeability [13], and the antihistamine cetirizine utilizes piperazine to modulate receptor affinity and pharmacokinetics [14]. Traditional anthelmintic preparations such as piperazine citrate rely on this scaffold to induce neuromuscular paralysis in parasites [15]. Thus, these examples show how inclusion of a piperazine unit contributes to potency, selectivity, and drug‐like properties across diverse pharmacological targets (Figure 1).

The saturated six‐membered heterocycle is a core motif exploited across multiple therapeutic areas. Its incorporation enables adjustment of molecular properties and target interactions, as exemplified by the anticancer kinase inhibitor imatinib, the antipsychotic dopamine–serotonin modulator aripiprazole, the antibacterial fluoroquinolone ciprofloxacin, and the antihistamine cetirizine. These structures show that diverse functionalization strategies around the piperazine ring support potency, selectivity, and favorable pharmacokinetic behavior.

Although piperazine is widely used, much of the research on piperazine remains dispersed across therapeutic classes and lacks a unified chemical perspective. Over the past decade, significant progress in synthetic methodology, structure–activity relationship (SAR) studies, and molecular modeling has deepened our understanding of how substitution patterns modulate biological outcomes [16, 17, 18]. Hybrid scaffolds combining piperazine with other heterocycles have shown combined effects, such as improved multitarget activity, enhanced blood‐brain barrier permeability, and reduced toxicity [19, 20]. Parallel advances in cheminformatics and machine learning enable predictive modeling of bioactivity, identification of conserved pharmacophores, and virtual screening of vast chemical libraries [17, 21, 22].

The integration of computational chemistry into piperazine‐based drug discovery is influencing design strategies. Recent approaches use data mining, molecular informatics, and unsupervised learning to correlate structural descriptors with biological outcomes [23, 24]. These approaches use large‐scale bioassay datasets and curated chemical fingerprints to build multidimensional representations of compound efficacy, enabling prioritization of candidates for in vitro and in vivo validation [8]. Machine learning and deep learning models are increasingly used to identify key physicochemical predictors of therapeutic success while reducing false‐positive discovery rates [11, 25]. Such approaches yield reproducible and interpretable models that capture essential variance in biological outcomes without oversimplifying chemical diversity [26]. These models extend beyond classification tasks, shaping future strategies in structure‐based drug design and AI‐driven compound optimization [27, 28]. Integration of these computational frameworks into decision‐support systems supports early‐stage candidate selection, reduces attrition in preclinical pipelines, and bridges the gap between medicinal chemistry and computational science [29]. Despite abundant preclinical leads, challenges in clinical translation persist, indicating the need for reliable target‐validation models and scalable synthetic routes. Integration of chemical knowledge and algorithmic modeling advances interdisciplinary innovation in drug design, particularly for piperazine‐derived scaffolds [30].

Cheminformatics and AI‐driven predictions accelerate candidate prioritization, their predictive capacity is constrained by training data, simplified assumptions, and limited representation of biological complexity. In silico outcomes should therefore be considered as supportive evidence that requires rigorous validation through enzymatic assays, cell‐based models, and preclinical pharmacology. Integration of computational approaches with experimental pipelines remains essential for achieving reliable translational outcomes.

Some reports on piperazine derivatives contains claims with limited experimental support. Multiple reports base structure–activity interpretations on narrow analog panels, which restricts generalization. Several studies present biological data from single assay conditions without confirmation across independent models. Several computational studies list predicted affinities without subsequent biological validation, which reduces confidence in reported conclusions. These issues highlight the need for a technical assessment of strengths and weaknesses within published work on this scaffold.

The goal of this review is to consolidate and critically evaluate the last decade's findings on piperazine chemistry and biology. It examines key synthetic strategies for constructing the piperazine core and its *N*‐substituted derivatives, summarizes SAR trends that link structural motifs to pharmacological activity, and highlights advances in their application across major therapeutic fields, including anti‐infective, neuroactive, anti‐inflammatory, and anticancer agents. It also examines future directions in green and sustainable synthetic methods, hybrid scaffold design, cheminformatics, and AI‐accelerated discovery [16]. By integrating chemical synthesis, SAR insights, and computational intelligence, this review aims to provide guidance for rational design of next‐generation piperazine‐based therapeutics.

## 2 Synthesis and Mechanistic Pathways of Piperazine Derivatives

Piperazine derivatives are widely synthesized using diverse chemical strategies that enable precise structural tuning and functional diversification for various therapeutic applications. Among the most fundamental methods is nucleophilic substitution, where the piperazine nucleus or its derivatives serve as nucleophiles and react with electrophilic alkyl dihalides or epoxides to yield *N,* *N*′‐disubstituted products. This classic SN2 pathway proceeds under mild conditions in polar aprotic solvents such as DMF or acetonitrile, often delivering yields above 80% [31, 32]. Another commonly employed method involves reductive amination of diamines, where diethylenetriamine or diethanolamine undergoes condensation with aldehydes or ketones in the presence of reducing agents such as NaBH_3_CN or catalytic hydrogenation. This approach allows for the synthesis of asymmetric piperazine derivatives with tailored substituents and has been applied in CNS‐active compound design [33, 34].

An alternative industrially relevant route is intramolecular cyclization of diethanolamine or diethylenetriamine derivatives under acidic or dehydrating conditions to directly generate the piperazine nucleus. This process is used in large‐scale synthesis due to its simplicity, low cost, and scalability [32]. For the preparation of aryl‐substituted derivatives, cross‐coupling reactions such as Suzuki–Miyaura and Buchwald–Hartwig aminations have become indispensable. These catalytic transformations use palladium complexes (e.g., Pd(PPh_3_)_4_ or Pd_2_(dba)_3_) with phosphine ligands and bases such as K_2_CO_3_ or Cs_2_CO_3_ in solvents like dioxane or toluene, typically producing yields in the range of 65%–90% [35].

### 2.1 *N*‐Monosubstitution of Piperazine: Strategies, Mechanisms, and Synthetic Applications

Selective *N*‐monosubstitution of piperazine remains an important transformation in heterocyclic medicinal chemistry, enabling the creation of asymmetric derivatives with optimized biological activity, pharmacokinetic behavior, and physicochemical profiles. The intrinsic challenge arises from the comparable nucleophilicity of the two nitrogen atoms in the piperazine ring, which often results in undesired bis‐alkylation. Over the last decade, numerous complementary methodologies have been developed to address this limitation and achieve regioselective functionalization with improved yields and reproducibility [3, 4].

Three principal strategies define current synthetic practice: protecting‐group‐assisted substitution, where one nitrogen atom is temporarily masked to control regioselectivity; stoichiometry‐controlled direct electrophilic alkylation, which exploits kinetic selectivity to favor monosubstitution; reductive amination, a stepwise approach involving iminium formation and subsequent reduction.

**SCHEME 1:**
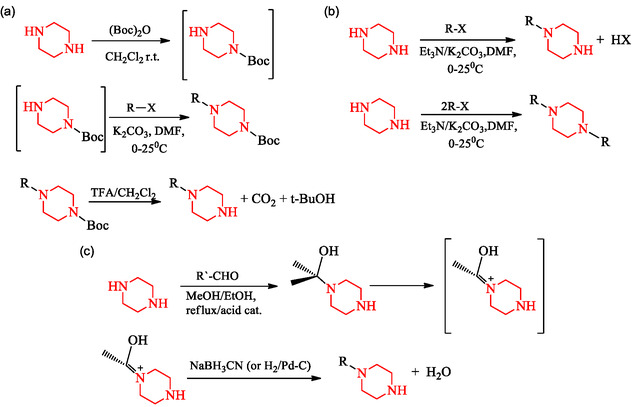
Overview of principal methods for *N*‐monosubstitution of piperazine: (a) Protecting‐group strategy using Boc; (b) direct electrophilic substitution under controlled stoichiometry; and (c) reductive amination pathway.

These complementary strategies are summarized in Scheme 1, which which outlines the principal routes.

Protecting‐group chemistry is among commonly used methods for achieving selective mono‐alkylation. In this approach, one nitrogen is deactivated by a bulky group such as tert‐butoxycarbonyl (Boc), making the second nitrogen selectively reactive. The typical sequence involves treatment of piperazine with Boc_2_O to yield *N*‐Boc‐piperazine, followed by alkylation with an electrophile (R—X) in the presence of a base (e.g., K_2_CO_3_ in DMF). Subsequent deprotection under acidic conditions (e.g., trifluoroacetic acid (TFA) in CH_2_Cl_2_) releases the mono‐alkylated product [33, 36, 37]. For instance, *N*‐(tert‐butoxycarbonyl)piperazine (NBPZ) has been synthesized by reacting piperazine with Boc_2_O, highlighting its utility in subsequent chemical modifications. In a study on fibrous metal adsorbents, *N*‐Boc‐piperazine was utilized in the amination step, demonstrating its role in controlled functionalization [38].

The field of piperazine chemistry has seen continued advances toward greener and more sustainable protection‐deprotection strategies, emphasizing methods that reduce reaction times and improve atom economy. Recent innovations in this area have focused on continuous‐flow approaches and mechanochemical techniques as methods aligned with these green chemistry principles. Continuous‐flow chemistry has emerged as a particularly useful area. A recent development is the use of a single‐step flow process for the deprotection of the tert‐butoxycarbonyl (Boc) protecting group using catalytic zinc‐hydroxyapatite columns. This method provides a benefit compared with conventional batch processes, which can require up to 24 h for complete deprotection and often lead to undesired side products necessitating extensive cleanup. By utilizing catalytic columns in a continuous‐flow setup, reaction times are significantly reduced, and waste generation is minimized, thereby enhancing the sustainability and atom economy of the process [39].

**SCHEME 2:**
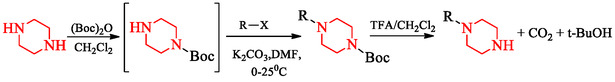
Detailed stepwise illustration of the Boc‐assisted mono‐alkylation route shown in Scheme 1a.

The synthetic sequence is illustrated in Scheme 2; the method involves (a) Boc protection of one nitrogen, (b) selective alkylation of the second, and (c) acid‐mediated deprotection.

**SCHEME 3:**
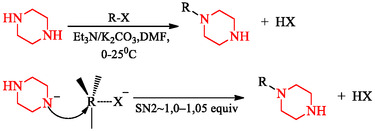
Direct electrophilic *N*‐alkylation of piperazine under controlled stoichiometry to afford monosubstituted derivatives.

An alternative approach circumvents protecting groups by controlling stoichiometry and reaction kinetics. Equimolar quantities of piperazine and an alkyl halide (R—X) under mild basic conditions (Et_3_N, DMF, 0°C–25°C) typically yield monosubstituted products, whereas excess electrophile or elevated temperature promotes bis‐substitution [40]. This atom‐economical strategy is attractive for industrial synthesis and scalable processes. Recent studies have also examined catalytic activation and transition‐metal mediation to further improve regioselectivity and functional‐group tolerance [41]. The general reaction course is shown in Scheme 3, showing the desired monosubstituted product and the competing bis‐alkylation pathway.

**SCHEME 4:**
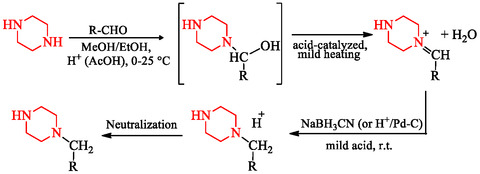
Reductive amination route for selective *N*‐monosubstitution. Piperazine condenses with an aldehyde to form an iminium intermediate, which is subsequently reduced to yield the secondary amine.

Reductive amination offers a useful route to *N*‐monoalkylated piperazines, especially for installing aryl–alkyl or heteroaryl–alkyl groups. The process involves condensation of piperazine with an aldehyde or ketone (R—CHO) to form a carbinolamine intermediate, which undergoes dehydration to yield an iminium species. Reduction using NaBH_3_CN or catalytic hydrogenation (H_2_/Pd–C) provides the final product [42]. Recent research has expanded this classical method through asymmetric organocatalytic and biocatalytic reductive amination, enabling enantioselective synthesis and greater structural diversity [41, 43]. Furthermore, metal‐catalyzed reductive amination protocols, including ruthenium‐ and iridium‐mediated approaches, have improved reaction efficiency, expanded substrate scope, and improved chemoselectivity [43, 44]. The standard synthetic route is shown in Scheme 4. The reductive amination route for *N*‐monosubstituted piperazines proceeds through a three‐step sequence involving condensation, dehydration, and reduction. In the first step, piperazine reacts with an aldehyde or ketone to form a carbinolamine intermediate, which subsequently undergoes acid‐catalyzed dehydration to yield an iminium ion. The iminium species is then reduced using sodium cyanoborohydride or catalytic hydrogenation to furnish the desired *N*‐alkylated piperazine. This protection‐free and selective process enables efficient incorporation of arylalkyl or heteroarylalkyl groups and can be extended through asymmetric or metal‐catalyzed variants to enhance selectivity and reaction efficiency.

Mechanistically, this reductive amination involves the nucleophilic addition of piperazine to a carbonyl compound, forming a carbinolamine intermediate, followed by acid‐catalyzed dehydration to an iminium ion and final reduction to yield the secondary amine. This stepwise transformation contributes to the selectivity and reaction efficiency of *N*‐monoalkylated piperazine synthesis [45, 46, 47].

Thus, these five complementary strategies, encompassing protecting‐group chemistry, stoichiometry‐controlled alkylation, and reductive amination, constitute a versatile synthetic toolbox for *N*‐monosubstitution of piperazine. Recent advances such as flow‐chemistry protocols, microwave‐assisted methods, metal‐catalyzed reductive transformations, and asymmetric biocatalytic approaches have significantly expanded the scope of this chemistry [43, 45, 48]. Continued integration of computational modeling and AI‐assisted reaction design is expected to further refine selectivity and efficiency, accelerating the development of next‐generation piperazine‐based therapeutics.

### 2.2 *N,* *N*′‐Disubstitution Strategies for Piperazine Derivatives

The synthesis of *N,* *N*′‐disubstituted piperazines is a an important step in medicinal chemistry, enabling fine‐tuning of molecular properties such as lipophilicity, metabolic stability, receptor selectivity, and target specificity [45, 49, 50]. Unlike monosubstitution, which deals with a single reactive site, disubstitution must control the reactivity of two equivalent nitrogen atoms. This challenge is further increased when the goal is to prepare unsymmetrical derivatives with two different substituents. Synthetic approaches are therefore categorized into symmetrical and unsymmetrical strategies, each with distinct methodologies and applications [51, 52].

#### 2.2.1 Symmetrical *N,N′*‐Disubstituted Piperazines

Symmetrical *N,* *N′*‐disubstituted piperazines, where both nitrogen atoms carry identical substituents, represent the most accessible and synthetically straightforward class of piperazine derivatives. These molecules are typically obtained through three fundamental strategies: direct alkylation, diacylation, and reductive amination, all of which exploit the nucleophilicity of the secondary amine nitrogen atoms and the ring's conformational flexibility.

In the direct alkylation approach, piperazine is treated with an excess of an alkyl halide (R—X) in the presence of a suitable base such as potassium carbonate (K_2_CO_3_) or sodium hydride (NaH), leading to the formation of *N,* *N*′‐dialkylpiperazines in a single step. This method remains a widely used route in early‐stage medicinal chemistry due to its simplicity and high atom economy. Similarly, diacylation reactions with acyl chlorides or anhydrides under basic conditions yield *N,* *N*′‐diacylpiperazines. The electrophilic acylating agent reacts with both nucleophilic nitrogen centers, producing symmetrical bis‐amide products with predictable yields and regioselectivity [42, 53].

Reductive amination offers an alternative and versatile route. Here, piperazine undergoes condensation with excess aldehyde or ketone to form a diiminium intermediate, which is subsequently reduced—typically using sodium cyanoborohydride (NaBH_3_CN) or hydrogenation over palladium—to produce *N,* *N′*‐dialkylpiperazines. This approach is particularly valuable in medicinal chemistry and combinatorial synthesis, where rapid generation of structural diversity is prioritized over site‐selectivity [43].

**SCHEME 5:**
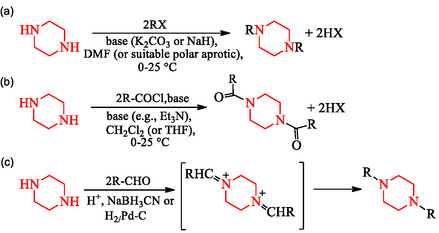
Synthetic and mechanistic strategies for symmetrical *N,* *N*′‐disubstitution of piperazine. Representative examples include: (a) Direct bis‐alkylation using excess alkyl halide; (b) diacylation with acyl chlorides or anhydrides; and (c) double reductive amination with aldehydes or ketones followed by reduction to yield *N,* *N′*‐dialkylated products.

The principal synthetic routes to symmetrical *N,* *N*′‐disubstituted piperazines are summarized in Scheme 5.

These methods form form an important basis of piperazine derivatization, enabling the preparation of libraries with significant structural and physicochemical diversity for drug discovery.

#### 2.2.2 Unsymmetrical *N,N′*‐Disubstituted Piperazines

Unsymmetrical *N,* *N′*‐disubstituted piperazines, where the two nitrogen atoms carry distinct substituents, are of interest in medicinal chemistry because they enable fine‐tuning of pharmacokinetic and physicochemical properties, increase selectivity, and allow structural diversification during lead optimization [54, 55, 56]. Their synthesis, however, is more challenging than that of symmetrical analogs because both nitrogen atoms exhibit comparable nucleophilicity, often resulting in statistical mixtures [57]. To overcome this, multistep synthetic strategies employing orthogonal protection or sequential functionalization are typically required [33].

**SCHEME 6:**
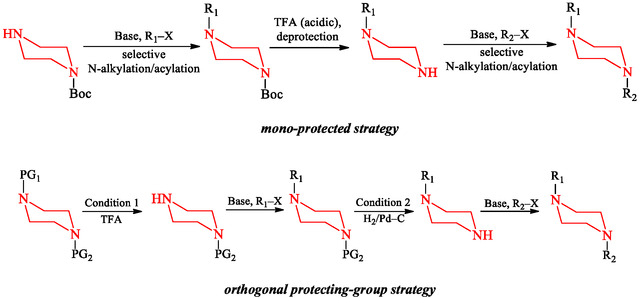
Synthetic approaches to unsymmetrical *N,* *N*′‐disubstituted piperazines via mono‐protected and orthogonal protecting‐group strategies.

A widely adopted approach involves mono‐protection and sequential alkylation (Scheme 6, route a). In this method, one nitrogen atom is temporarily protected with a group such as Boc to suppress its reactivity [52]. The free nitrogen is then alkylated or acylated with the first substituent (R_1_) under basic conditions [58]. Subsequent acid‐mediated deprotection (commonly using TFA) regenerates the second amine, which is subsequently functionalized with a different electrophile (R_2_–X or R_2_–COCl) to yield the final unsymmetrical product bearing N–R_1_ and N–R_2_ groups [59]. This strategy is synthetically straightforward and widely used for library synthesis, although it requires careful control of reaction conditions to prevent over‐alkylation [60].

An alternative and flexible strategy employs orthogonal protecting groups (Scheme 6, route b), which enable independent manipulation of both nitrogen atoms [57, 61]. In this approach, each nitrogen is protected with a distinct protecting group (PG_1_ and PG_2_) that can be selectively removed under orthogonal conditions [62]. Stepwise deprotection allows sequential introduction of R_1_ and R_2_ without cross‐reactivity, thereby increasing regioselectivity and synthetic flexibility [63]. Orthogonal strategies are particularly valuable when designing complex molecules or when late‐stage diversification is desired for SAR exploration [64]. In these orthogonal approaches, the terms “Condition 1 and Condition 2” (as indicated in Scheme 6) refer to the selective removal of each protecting group under mutually exclusive chemical environments [65]. Typically, Condition 1 involves mild acidic treatment (e.g., TFA) to cleave acid‐labile groups such as Boc, while leaving groups like Cbz or Alloc intact. Condition 2 then removes the second protecting group, for example, via catalytic hydrogenolysis (H_2_/Pd–C) for Cbz or palladium‐catalyzed allyl cleavage for Alloc—without affecting the first substitution [52, 61, 63]. This selective sequence improves control over substitution and enables preparation of complex derivatives.

The choice between mono‐protection and orthogonal strategies depends on the target complexity, scalability, and downstream modifications. Mono‐protected routes are generally faster and well‐suited to early‐stage drug discovery [66], whereas orthogonal approaches provide stronger control and are preferred when synthetic precision and regioselectivity are essential [67].

Thus, the synthesis of unsymmetrical *N,* *N′*‐disubstituted piperazines is more complex than that of their symmetrical counterparts due to the similar nucleophilicity of both nitrogen atoms, which necessitates multistep strategies to achieve selective functionalization. Mono‐protection followed by sequential substitution provides an effective route for compound library generation, while reaction control is important to limit over‐alkylation. In contrast, orthogonal protecting‐group strategies provide precision by enabling stepwise substitution through selective deprotection under distinct chemical environments, commonly referred to as Condition 1 and Condition 2. These approaches not only allow for precise regioselective functionalization but also enhance the chemical diversity and pharmacological tunability of piperazine scaffolds, establishing them as important tools in contemporary medicinal chemistry and lead optimization.

### 2.3 Modern Synthetic Methodologies

**SCHEME 7:**
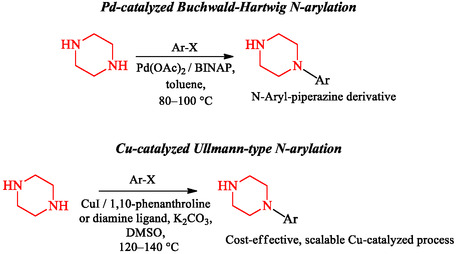
Cross‐coupling strategies for piperazine functionalization.

Current methods for piperazine synthesis focus on efficiency, selectivity, and scalability. Advanced synthetic strategies improve reaction performance, broaden substrate range, and support downstream use. Among these, transition‐metal‐catalyzed cross‐coupling, microwave‐assisted organic synthesis (MAOS), and continuous‐flow chemistry have become widely used approaches, each offering specific advantages for drug discovery and process chemistry [33, 34, 62, 68]. The Buchwald–Hartwig amination and Ullmann‐type *N*‐arylation reactions have become important tools for constructing *N*‐aryl and *N*‐heteroaryl piperazine derivatives (Scheme 7). The Buchwald–Hartwig methodology, which employs palladium catalysts (e.g., Pd(OAc)_2_ or Pd_2_(dba)_3_), biaryl phosphine or NHC ligands, and strong bases such as NaOtBu, allows efficient coupling of aryl halides with secondary amines under mild conditions [33, 34, 62, 68]. This reaction is characterized by good functional‐group tolerance, rapid kinetics, and suitability for late‐stage diversification, enabling direct modification of advanced drug candidates [62, 68]. The Ullmann reaction, employing copper catalysts and diamine ligands, provides a cost‐effective and scalable alternative for similar transformations, albeit often requiring higher temperatures and longer reaction times [69]. Despite these limitations, improved ligand systems and heterogeneous catalysts have expanded substrate scope and improved catalytic efficiency, making Ullmann coupling highly relevant for industrial‐scale processes [69, 70].

**SCHEME 8:**
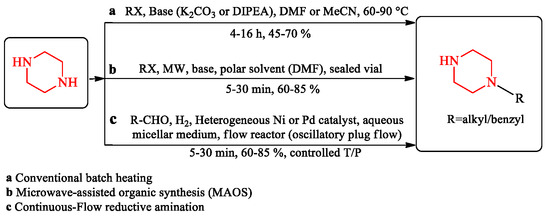
Comparative synthetic platforms for mono‐*N*‐alkylation of piperazine. (a) Conventional batch heating. (b) Microwave‐assisted organic synthesis (MAOS). (c) Continuous‐Flow reductive amination.

Microwave irradiation accelerates reaction rates and can improve yields in synthetic methodology. Flow‐based systems provide control over reaction parameters and enable safe scale‐up from milligram to kilogram quantities. Unlike conventional conductive heating, microwaves provide rapid, volumetric energy transfer, which reduces reaction times from several hours to minutes while minimizing side‐product formation [71, 72]. In piperazine chemistry, MAOS has been applied to *N*‐alkylation, *N*‐acylation, and reductive amination reactions, enabling rapid library synthesis with improved reproducibility [72, 73, 74]. This acceleration makes MAOS valuable in SAR campaigns and parallel medicinal chemistry, where rapid iterations of chemical space are required. Microwave conditions often allow reactions to proceed under milder conditions or with reduced catalyst loading, supporting principles of green chemistry and sustainability [71]. Continuous‐flow synthesis has changed the preparation of piperazine derivatives by enabling precise control over reaction parameters, enhancing safety, and simplifying scale‐up from milligram to kilogram quantities [70, 75]. Flow systems are effective at handling reactive intermediates and hazardous reagents, allowing processes such as sequential alkylation, cross‐coupling, and reductive amination to proceed with high reproducibility and improved process safety [75, 76]. Recent advances include oscillatory plug‐flow reactors and micellar flow systems, which further improve reaction efficiency and reduce solvent usage [75]. Furthermore, catalytic continuous‐flow reductive amination has been demonstrated under hydrogen, offering a greener and scalable route to complex piperazine derivatives [76]. Scheme 8 compares conventional, microwave‐assisted, and continuous‐flow platforms for mono‐*N*‐alkylation of piperazine. It illustrates how microwave heating (MAOS) and flow chemistry accelerate reaction kinetics and improve yields relative to traditional batch conditions. Microwave irradiation achieves 60%–85% yields within minutes under mild temperatures, whereas flow systems provide precise control of temperature, pressure, and residence time for scalable synthesis. These approaches represent greener, high‐throughput strategies that enhance both reaction efficiency and reproducibility in piperazine functionalization. Scheme 8 depicts a continuous flow chemistry setup for the synthesis of piperazine compounds. Starting materials and reagents are combined and introduced into a pump, which feeds them into a flow reactor where the chemical transformation occurs under controlled conditions. The flow reactor ensures efficient mixing, precise temperature control, and optimized reaction time, leading to the formation of the desired piperazine product. This approach offers advantages in scalability, safety, and reaction repeatability compared to traditional batch synthesis, making it relevant for modern synthetic methodologies in piperazine chemistry.

The scope, mechanism, and representative examples of these modern methodologies are summarized in Table 1, which highlights their synthetic utility and applicability to piperazine chemistry.

**TABLE 1:** Comparative overview of modern synthetic approaches to piperazine functionalization.

Method	Key reagents/Catalysts	Typical time/ Temperature	Scalability & applications	Reference
Buchwald–Hartwig (Pd) *N*‐arylation	Pd catalyst (e.g., Pd_2_(dba)_3_); biaryl phosphine or NHC ligand; NaOtBu; Ar—X; polar solvent	1–8 h; 60°C–110°C	Broad scope for *N* *‐*aryl/heteroaryl piperazines; ideal for late‐stage diversification and parallel synthesis	[1, 2, 3]
Ullmann (Cu) *N*‐arylation	CuI/CuBr; diamine ligands; Ar—Br/I; K_2_CO_3_/Cs_2_CO_3_; DMF/DMSO	4–24 h; 80°C–140°C	Cost‐effective and robust; excellent for heteroaryl partners; scalable	[4, 5]
MAOS	Alkyl halides, acyl chlorides, or aldehydes/ketones; NaBH_3_CN or Pd/H_2_; polar solvent	5–30 min; 60°C–140°C	Rapid, high‐yield, clean; ideal for library synthesis; limited by reactor volume	[6, 7, 8, 9]
Flow reductive amination	R—CHO/ketone; piperazine; NaBH_3_CN or H_2_ over Pd/C; microreactor	Seconds–minutes; 25°C–120°C	Excellent scalability and safety; reproducible and easily automated	[5, 10, 11]
Flow cross‐coupling	Pd or Cu catalysts; Ar—X; ligand; base; back‐pressure control	Minutes–hours; 60°C–130°C	Continuous production, minimal catalyst waste, and easy integration	[2, 3, 4, 5, 10, 11]

Modern methodologies such as classical *N*‐monosubstitution, *N,* *N*′‐disubstitution, transition‐metal‐catalyzed cross‐coupling, MAOS, and continuous‐flow processing form a set of methods for modern piperazine chemistry. These approaches accelerate scaffold diversification, enable controlled modulation of physicochemical properties, and support scalable synthesis of complex piperazine derivatives. The incorporation of cross‐coupling, MAOS, and flow techniques represents a shift in heterocyclic synthesis by improving reaction efficiency, expanding structural diversity, and supporting sustainable translation from laboratory research to industrial production. Transition‐metal catalysis allows late‐stage functionalization, MAOS reduces reaction time and increases throughput, while flow chemistry provides fine control of process parameters and scalability. These methods form a platform for piperazine synthesis and support the design of new drug candidates.

### 2.4 C—H Functionalization of the Piperazine Ring

Direct C—H functionalization of saturated nitrogen heterocycles such as piperazine provides access to analogs that are less accessible to obtain through classical *N*‐functionalization routes [59, 77, 78, 79]. Unlike conventional derivatization, which requires prefunctionalized starting materials, C—H activation enables late‐stage modification of the carbon backbone, creating new positions for molecular elaboration and enabling fine‐tuning of pharmacokinetic and pharmacodynamic properties [80, 81]. This approach has proven useful in medicinal chemistry, where subtle changes to the piperazine substitution pattern can significantly influence metabolic stability, receptor selectivity, and solubility without altering the core scaffold [82, 83, 84].

A key advance in this field has been the use of transition‐metal‐catalyzed C—H activation to selectively cleave otherwise inert C—H bonds. Strategies based on palladium(II), rhodium(III), and ruthenium(II) catalysts have enabled direct arylation, alkylation, and alkenylation of the piperazine framework under mild conditions, often with high regioselectivity [77, 78, 85]. For example, Pd(II)‐catalyzed arylation allows direct installation of aryl groups at the C‐2 or C‐3 positions, expanding chemical diversity beyond what is achievable with prefunctionalized substrates [82, 83]. In parallel, photoredox‐mediated radical alkylation has emerged as a useful complementary approach, using visible light and photocatalysts (e.g., Ir or Ru complexes) to achieve alkyl introduction under mild, redox‐neutral conditions [80, 81].

**SCHEME 9:**
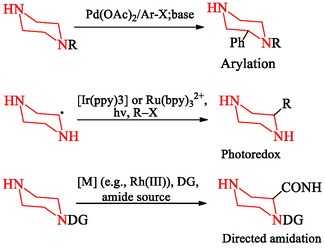
Representative C—H functionalization strategies of piperazine.

Recent advances have also used directing‐group strategies, in which coordinating auxiliaries guide the catalyst to specific C—H sites, thereby overcoming challenges of regioselectivity [78, 79]. Bidentate directing groups have enabled selective C(sp^3^)—H amidation and fluorination, delivering functional handles for subsequent diversification [86, 87]. These methodologies are used in multiple studies for late‐stage modification of drug‐like piperazines, enabling rapid analog generation for SAR studies and medicinal chemistry optimization [83, 86]. The three major mechanistic classes of piperazine C—H functionalization are illustrated in Scheme 9, which shows complementary strategies based on transition‐metal catalysis, photoredox radical generation, and directing‐group‐assisted transformations.

A schematic overview of advanced approaches for direct modification of the piperazine carbon framework. (Top) Pd(II)‐catalyzed C—H arylation enables regioselective installation of aryl groups at the piperazine backbone [77, 78, 79, 82, 83, 85]. (Middle) Photoredox‐catalyzed alkylation uses visible light and photocatalysts to achieve late‐stage alkylation under mild conditions [80, 81]. (Bottom) Directed amidation uses coordinating groups to guide selective C—H activation, yielding valuable amide‐functionalized products for further synthetic elaboration [84, 86].

Despite these advances, limitations remain. Achieving precise site‐selectivity remains challenging, particularly for complex scaffolds with multiple reactive sites [77, 78, 82]. The reliance on preinstalled directing groups can increase synthetic complexity and reduce atom economy, while high catalyst costs and limited scalability restrict industrial adoption. However, progress in ligand design, earth‐abundant catalysts, electrochemical activation, and photochemical methodologies is addressing these challenges. As these approaches mature, C—H functionalization is anticipated to become a mainstream platform for late‐stage diversification, complementing traditional *N*‐functionalization and cross‐coupling strategies in medicinal chemistry and process development [83, 85].

Several synthetic reports demonstrate reactions on simplified substrates that do not represent the complexity of substituted piperazines used in medicinal design. Reaction performance is often shown with a limited set of examples, which restricts evaluation of functional‐group tolerance. Several protocols employ high catalyst loadings or costly metals without discussion of practical feasibility. These factors limit the reliability of claims regarding broad synthetic utility.

A summary of representative C—H functionalization methodologies applied to piperazine derivatives, including catalysts, directing strategies, and reaction outcomes, is provided in Table 2.

**TABLE 2:** Representative C—H functionalization strategies for piperazine derivatives.

Method	Catalyst/Reagents	DG (typical)	Conditions	Selectivity/ Transformation	Notes	Reference
Pd(II)‐catalyzed C(sp^3^)—H arylation	Pd(OAc)_2_; (often) carboxamide‐, picolinamide‐, or 8‐AQ–type DG; aryl iodide/boronate; base	Bidentate amide/ picolinamide/8‐AQ	80°C–120°C; polar aprotic solvent; O_2_ or Ag(I) not always required	β‐ to γ‐C(sp^3^) arylation on saturated aza‐rings (piperidine/pyrrolidine); high regio‐ and diastereoselectivity with DG control	Broadest precedent on piperidines; strategies translate to piperazine with appropriate DG installation	[78, 87, 88]
Rh(III)‐catalyzed directed C(sp^3^)—H amidation (dioxazolones)	[Cp*RhCl_2_]_2_/AgSbF_6_; dioxazolone (R—C(O)—N=O)	Bidentate auxiliaries (e.g., amide, dithiane‐tethered)	50°C–100°C; DCE/MeCN; additive (carboxylate)	β‐C(sp^3^) amidation under mild, oxidant‐lean conditions	Dioxazolone nitrene transfer; handles complex scaffolds; DG removable	[89, 90, 91]
Photoredox C—H alkylation (HAT/radical)	Ir(ppy)_3_ or Ru(bpy)_3_; organoradical precursors (e.g., alkyl bromides, carboxylates); light (blue/green)	Often none; *N*‐protection or electronic bias in piperazine	RT; visible light; mild bases	Site‐selective α/β‐C(sp^3^) alkylation on piperazine ring	Direct piperazine precedent; late‐stage diversification; predictable site‐selectivity by differentiating the two nitrogens	[59, 92, 93]
Cu‐catalyzed C(sp^3^)—H functionalization/ halogenation‐initiated LSF	Cu(I)/Cu(II) salts; NFSI or Selectfluor (oxidant); alcohol/H_2_O or nucleophiles downstream	Often none; substrate bias (tertiary amine)	Mild (rt‐60°C); CDC/oxidation to iminium/hemiaminal then capture	α‐C(sp^3^) hydroxylation/alkoxylation; platform for halogenation/fluorination and further C—X or C—C	General LSF platform on *N*‐heterocycles (incl. piperidine/piperazine motifs in scope tables); operationally simple	[94]
Directed C(sp^3^)—H fluorination	Pd(OAc)_2_; Selectfluor; amide‐type DG	Bidentate amide/ sulfonamide	60°C–100°C; polar solvent	Directed methylene fluorination at α/β positions	Powerful but substrate/DG specific; complements Cu/NFSI LSF above	[95, 96]
Electrochemical C(sp^3^)—H functionalization (oxidation‐driven LSF)	Graphite/BDD anode; quinuclidine or NHPI mediators (var.); undivided cell	Often none; tertiary amine substrates	RT; constant current; MeCN or HFIP mixtures	α‐C(sp^3^) oxygenation/alkoxylation/azidation; scalable and green	Amenable to complex saturated *N*‐heterocycles**;** pairs with photoredox (“electro‐photochemical”)	[97, 98, 99]

**FIGURE 2:**
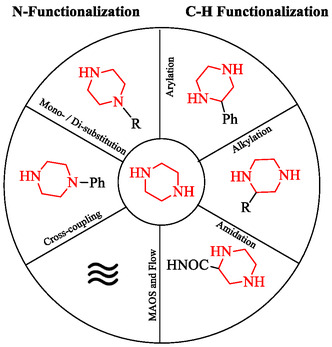
Complementary synthetic strategies for piperazine diversification. Classical *N*‐functionalization approaches (left), including *N*‐monosubstitution, *N,* *N′*‐disubstitution, cross‐coupling, MAOS, and flow chemistry, enable efficient modification at the nitrogen atoms. C—H functionalization methods (right), such as arylation, alkylation, and amidation, directly remodel the carbon skeleton, introducing new vectors for structural diversity.

The synthetic field of piperazine chemistry has advanced from classical *N*‐functionalization strategies to advanced methodologies that enable controlled molecular design. Approaches such as *N*‐monosubstitution, *N,* *N*′‐disubstitution, transition‐metal‐catalyzed cross‐coupling, microwave‐assisted synthesis, and flow chemistry have expanded the scope, efficiency, and scalability of nitrogen‐centered transformations, facilitating the efficient generation of diverse libraries for medicinal chemistry applications. Complementary to these, recent advances in C—H functionalization provides a effective means to directly modify the carbon framework, introducing additional vectors for chemical diversity and three‐dimensional diversification that were not attainable through nitrogen substitution alone. Thus, these methodologies establish an integrated and complementary toolbox (Figure 2) that enables chemists to access greater structural complexity and to adjust the pharmacological and physicochemical profiles of piperazine‐based scaffolds for future drug discovery.

## 3 Mechanism of Action of Piperazine‐Based Compounds on Cells

Piperazine‐containing compounds exhibit a broad range of biological effects by interacting with diverse cellular targets and modulating essential biochemical pathways. Their distinct conformational flexibility, protonatable nitrogen centers, and tunable physicochemical properties make them adaptable scaffolds for drug‐target interactions across multiple therapeutic domains [77, 78, 79]. A fundamental mechanism involves binding to G‐protein‐coupled receptors (GPCRs), such as dopamine D_2_, serotonin (5‐HT_1_A/_2_A), and histamine receptors, where piperazine moieties act as basic anchors that enhance ligand‐receptor affinity and modulate downstream signaling [100, 101]. This property is central to the activity of many neuroactive compounds, including antipsychotics and antidepressants, where selective receptor engagement governs therapeutic efficacy [82]. Beyond receptor modulation, enzyme inhibition is another major mechanism. Piperazine derivatives can form noncovalent interactions within enzyme active sites, often mimicking transition‐states to block catalytic activity [102]. Such inhibition is relevant in targeting tyrosine kinases, proteases, and topoisomerases, which are critical regulators of cell proliferation and survival [103]. In antimicrobial contexts, similar interactions with bacterial enzymes underlie the efficacy of piperazine‐containing fluoroquinolone derivatives [80].

Piperazine‐based molecules also influence cellular transport processes, notably by modulating neurotransmitter transporters such as those for serotonin, dopamine, and GABA [1, 2, 3, 104]. This reuptake inhibition mechanism underlies the clinical effects of anxiolytic and antidepressant agents such as buspirone and trazodone [105]. Additionally, many piperazine analogs regulate ion‐channel function, modifying calcium and sodium fluxes to modulate neuronal excitability—a mechanism exploited in the treatment of epilepsy and migraine [106, 107]. The amphiphilic nature of certain piperazine derivatives facilitates membrane interactions, increasing permeability, altering membrane potential, and enabling selective accumulation in specific cellular compartments [6]. These physicochemical effects contribute to improved drug delivery and selective cytotoxicity in cancer cells. Moreover, several piperazine‐containing molecules can induce apoptosis by disrupting mitochondrial function or activating caspase cascades [108], while others inhibit cell proliferation through cell cycle arrest via cyclin‐dependent kinase (CDK) modulation [109].

A comprehensive overview of these mechanisms, their biological targets, representative examples, and therapeutic applications is presented in Table 3.

**TABLE 3:** Mechanisms of action of piperazine‐based compounds: cellular targets, effects, and therapeutic relevance.

Mechanism of action	Cellular target	Molecular effect	Representative compound	Therapeutic area	Reference
GPCR binding/Modulation	Dopamine D_2_, 5‐HT_1_A/_2_A, H_1_ receptors	Agonism or antagonism modulating neurotransmission	Aripiprazole, risperidone	Antipsychotic, antidepressant	[110]
Enzyme inhibition	Tyrosine kinases, topoisomerases, proteases	Noncovalent inhibition of catalytic activity	Imatinib derivatives, ciprofloxacin analogs	Oncology, antimicrobial	[19]
Transporter modulation	Serotonin, dopamine, GABA transporters	Inhibition of neurotransmitter reuptake	Buspirone, trazodone	Neuroactive disorders, anxiety	[111, 112]
Ion channel regulation	Voltage‐gated Ca^2+^/Na^+^ channels	Modulation of ion flux, neuronal excitability	Flunarizine, lomerizine	Migraine, epilepsy	[113]
Membrane interaction/Permeabilization	Phospholipid bilayers	Enhanced permeability, altered membrane potential	Piperazine‐based amphiphiles	Antimicrobial, anticancer	[107]
Apoptosis induction	Mitochondrial membrane, Caspase cascade	Activation of programmed cell death pathways	Piperazine‐thiourea hybrids	Anticancer	[114]
Cell cycle arrest/Proliferation inhibition	Cyclin‐dependent kinases (CDKs)	Blockade of G_1_/S or G_2_/M transitions	Piperazine‐pyrimidine hybrids	Oncology	[26]

## 4 Anti‐Infective Applications of Piperazine Derivatives

### 4.1 Antiparasitic Activity

**FIGURE 3:**
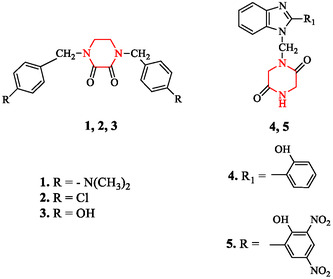
Piperazine‐2,3‐dione and piperazine‐2,5‐dione derivatives.

Piperazine derivatives remain widely used in the treatment of parasitic diseases, especially helminthiasis. Traditional formulations such as piperazine citrate are effective against *Ascaris lumbricoides* and related roundworms and continue to play a role in veterinary and human therapy [15]. Simple scaffolds such as piperazine‐2,3‐dione and piperazine‐2,5‐dione (Figure 3) have been investigated as useful cores for the design of antiparasitic leads. These dione derivatives provide rigid backbones that can be functionalized at different positions, creating analogs with improved pharmacological stability [3, 115]. They serve as chemical precursors for later hybrid designs, including benzimidazole‐linked compounds with higher potency.

**FIGURE 4:**
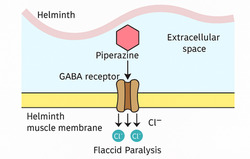
Mechanism of action of piperazine in helminths: Binding to GABA receptors induces chloride ion influx, causing flaccid paralysis.

The mechanism of action is well established. Piperazine compounds act as GABA‐receptor agonists in nematodes, causing chloride influx, hyperpolarization of neuronal membranes, and flaccid paralysis of the parasite (Figure 4) [116]. This neuromuscular blockade prevents worms from maintaining their position within the intestine, resulting in expulsion.

**FIGURE 5:**
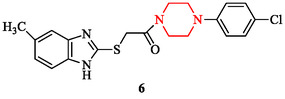
Structure of 1‐(4‐(4‐chlorophenyl)piperazin‐1‐yl)‐2‐((5‐methyl‐1H‐benzo[d]imidazol‐2‐yl)thio)ethenone.

Recent studies confirm the value of structural hybrids. Benzimidazole‐piperazine conjugates have shown high activity against *Trichinella spiralis* larvae. One example, 1‐(4‐(4‐chlorophenyl)piperazin‐1‐yl)‐2‐((5‐methyl‐1H‐benzo[d]imidazol‐2‐yl)thio)ethanone (Figure 5), achieved more than 90% inhibition of larval activity at 100 µg/mL in vitro [117].

Computational methods have expanded discovery in this field. Quantitative SAR (QSAR) libraries and molecular docking studies highlight nitrogen substitution and aromatic modifications as key for potency [118]. Artificial intelligence is also emerging in parasite drug discovery workflows [119]. These approaches may reduce resistance development and accelerate design of next‐generation antiparasitic agents.

### 4.2 Antimicrobial Activity

**FIGURE 6:**
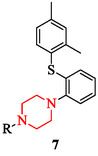
1‐(2‐(2,4‐Dimethylphenylthio)phenyl)piperazine.

**FIGURE 7:**
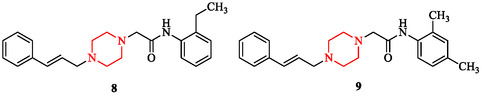
Derivatives of 1‐[(E)‐3‐phenyl‐2‐propenyl]piperazine.

Piperazine scaffolds display notable activity against bacterial and fungal pathogens. Their effects are determined by structural substitution, hybrid design, and physicochemical tuning rather than by generic membrane disruption. SAR studies indicate that lipophilic aryl and thioaryl groups enhance bacterial penetration. The thioaryl‐substituted analog 1‐(2‐(2,4‐dimethylphenylthio)phenyl)piperazine (Figure 6) demonstrated potent inhibition of *Staphylococcus aureus*, *Pseudomonas aeruginosa*, and *Escherichia coli*, highlighting the impact of lipophilic substitution [3, 40]. Side‐chain extension also influences potency. Cinnamyl derivatives (compounds 8, 9) of 1‐[(E)‐3‐phenyl‐2‐propenyl]piperazine (Figure 7) illustrate how π‐extended substituents improve Gram‐negative activity by enhancing outer‐membrane traversal and enzyme binding [19, 40].

Recent studies by Khaiitova et al. [120] also demonstrated that newly synthesized modified piperazine derivatives exhibit notable local anesthetic and antibacterial activity, indicating the pharmacological utility of this scaffold in both peripheral and systemic models.

**FIGURE 8:**
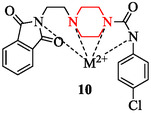
Metal complexes of 4‐(2‐(1,3‐dioxoisoindolin‐2‐yl)ethyl)‐*N*‐phenylpiperazine‐1‐carboxamide.

Another approach involves metal complexation, which modifies the electronic profile and drug uptake. Metal complexes of 4‐(2‐(1,3‐dioxoisoindolin‐2‐yl)ethyl)‐*N*‐phenylpiperazine‐1‐carboxamide (compound 10) synthesized by the authors in [121], incorporating metal ions such as Cu^2+^, Zn^2+^, Co^2+^, and Ni^2+^ also exhibited antibacterial activity against methicillin‐resistant *S. aureus* (MRSA) (Figure 8). Among them, the copper‐based complex (5Cu) showed highest activity antibacterial effect. The minimum inhibitory concentration (MIC) of the copper complex against MRSA was reported as 30 ± 0.15 µg/mL and 20 ± 0.12 µg/mL, with corresponding inhibition zones of 14.5 ± 0.04 mm and 15 ± 0.08 mm, respectively, comparable to the standard antibiotics streptomycin and bacitracin (10 µg/mL).

Published antimicrobial studies often present minimal strain diversity and limited assay standardization. Some articles list MIC values without reporting reference controls, which limits comparison across studies. A part of the reported activity is based on preliminary screening without verification in resistant strains or biofilm systems, which limits interpretation of therapeutic potential. These constraints reduce the reliability of several activity claims in the literature.

**FIGURE 9:**
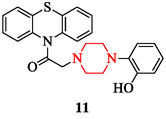
2‐(4‐(2‐Hydroxyphenyl)piperazin‐1‐yl)‐1‐(10H‐phenothiazin‐10‐yl)ethenone.

Hybrid designs with fused heterocycles also influence biological response. The compound 2‐(4‐(2‐hydroxyphenyl)piperazin‐1‐yl)‐1‐(10H‐phenothiazin‐10‐yl)ethanone (compound 11), synthesized by Iranian researchers, was investigated for antibacterial, antifungal, and antitubercular activity [122] (Figure 9). Antibacterial testing revealed strong effectiveness, particularly against Gram‐positive organisms such as *S. aureus* and *Bacillus subtilis*. Its antibacterial activity against *B. subtilis* was found to be comparable to that of the reference drug streptomycin. Notably, the compound showed no detectable inhibitory zones against Gram‐negative bacteria.

Given the success of piperazine‐fluoroquinolone hybrids, research should also evaluate combinations with efflux pump inhibitors to overcome multidrug resistance [123, 124].

In fungal pathogens, hybridization with azole motifs has emerged as a effective strategy. Piperazine‐azole conjugates inhibit ergosterol biosynthesis and retain solubility, leading to marked inhibition of *Candida albicans* and *Aspergillus fumigatus* [19, 42]. Substituents such as pyridyl and fluorinated groups increase metabolic stability and extend target residence time [2]. Electron‐donating groups at the piperazine nitrogen improve selectivity, while bulky lipophilic moieties often reduce solubility and limit oral absorption. These findings provide guidance for scaffold optimization.

Several piperazine‐based compounds have been tested with promising results. One derivative showed high inhibition of *C. albicans* by inducing membrane disruption, with an IC_50_ value of 2.5 µM. Another analog targeting *A. fumigatus* interfered with ergosterol and exhibited an IC_50_ of 3.8 µM. These findings confirm that piperazine scaffolds can yield potent antifungal agents, especially in settings of rising resistance to traditional azoles and polyenes. Reports on alkylated piperazines and piperazine‐azole hybrids demonstrated comparable potency, validating the potential of these designs for antifungal therapy. At the same time, resistance trends in fungal pathogens highlight the need of developing new chemical classes [125, 126].

Recent synthetic work has generated structurally modified piperazine derivatives that enhance potency and selectivity by either disrupting fungal lipid bilayers or blocking sterol metabolic pathways [2, 42]. Molecular dynamics and structural biology studies have clarified the membrane‐disruptive properties of piperazine‐azole hybrids and their binding at sterol 14α‐demethylase, supporting rational design of agents that can penetrate fungal biofilms [127]. These insights position the piperazine scaffold as a promising foundation for next‐generation antifungal drugs.

Clinical translation, however, remains limited. Failures often arise from resistance in Gram‐negative bacteria, poor solubility, or CYP‐mediated drug–drug interactions in antifungal therapy. Current strategies to improve translation include the development of metal‐assisted analogs that extend Gram‐negative coverage, hybrid scaffolds with enhanced biofilm activity, and coadministration with efflux pump inhibitors [2, 3, 40, 42].

### 4.3 Neuroactive Applications

Piperazine derivatives represent an important class of neuroactive agents with therapeutic potential in epilepsy, depression, and psychosis. Their dual nitrogen atoms and flexible scaffold allow modulation of multiple neurotransmitter systems and ion channels, leading to broad pharmacological outcomes.

**FIGURE 10:**
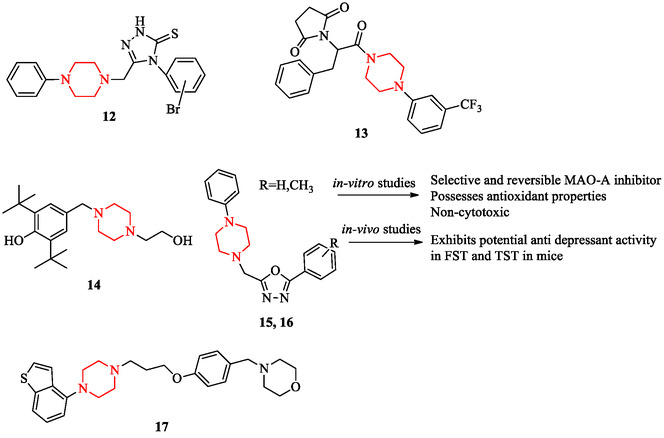
Representative piperazine‐based neuroactive scaffolds: Anticonvulsant derivatives, antidepressant candidates, and antipsychotic agent.

In the field of epilepsy, structurally modified piperazine derivatives have demonstrated notable anticonvulsant properties. Compounds bearing triazole or thiadiazole fragments and phenylpiperazine amides inhibited electroshock‐induced seizures and displayed broad‐spectrum antiepileptic activity in animal models (Figure 10, compounds 12, 13) [128, 129]. These findings are supported by advances in the identification of epilepsy biomarkers that confirm ion‐channel and neurotransmitter‐related mechanisms [130]. SAR studies highlight that electron‐donating aromatic substituents and heteroaryl linkers improve seizure‐suppressive activity by stabilizing sodium and calcium channel function.

In mood disorder research, the hydroxyethyl‐substituted derivative LQFM212 and oxadiazole‐piperazine hybrids have shown antidepressant‐like activity through modulation of monoaminergic pathways and inhibition of monoamine oxidase‐A (Figure 10, compounds 14–16) [131, 132]. Behavioral studies suggest that these compounds enhance neurotrophic signaling, further supporting their therapeutic potential [131]. Broader reviews confirm that arylpiperazine scaffolds remain a central class for developing antidepressant candidates with polypharmacological activity [133]. SAR analyses indicate that aromatic substitution at the N‐4 position of piperazine with electron‐rich heterocycles confers strong enzyme inhibition and favorable monoaminergic binding.

Promising antipsychotic profiles have also been reported for newly synthesized arylpiperazine derivatives. A representative multireceptor antagonist reduced schizophrenia‐like hyperlocomotion and improved cognition in preclinical models (Figure 10, compound 17) [134]. The clinical potential of arylpiperazine derivatives is reinforced by both medicinal chemistry surveys [133] and radioligand imaging studies showing their suitability for targeting central nervous system receptors [135]. These findings support the SAR principle that bulky aromatic substituents at the N‐4 position facilitate multitarget engagement with dopamine and serotonin receptors, improving efficacy while limiting extrapyramidal effects.

Overall, these studies underscore the therapeutic versatility of the piperazine scaffold in neuroactive disorders. Figure 10 illustrates the structural diversity of piperazine‐based neuroactive compounds spanning anticonvulsant, antidepressant, and antipsychotic domains. Their ability to integrate ion‐channel regulation with monoaminergic modulation positions them as adaptable templates for epilepsy, depression, and psychosis. Future work should combine receptor‐binding assays, EEG‐based seizure profiling, and neuroimaging (PET/SPECT) to refine translational validity and optimize safety profiles.

### 4.4 Anti‐Inflammatory Activity

Piperazine derivatives have received growing interest for their anti‐inflammatory potential, complementing well their known roles in antimicrobial and neuroactive chemistry. The piperazine scaffold's synthetic flexibility allows substitution patterns that can interact favorably with inflammatory mediators, such as cyclooxygenases, lipoxygenases, cytokines, and reactive oxygen species. A recent review of the medicinal chemistry of piperazines highlights their emerging relevance in modulating inflammatory pathways, and reports that compound 80a achieved a 68.02% inhibition in an anti‐inflammatory assay, illustrating how subtle structural changes can tune potency [5].

**FIGURE 11:**
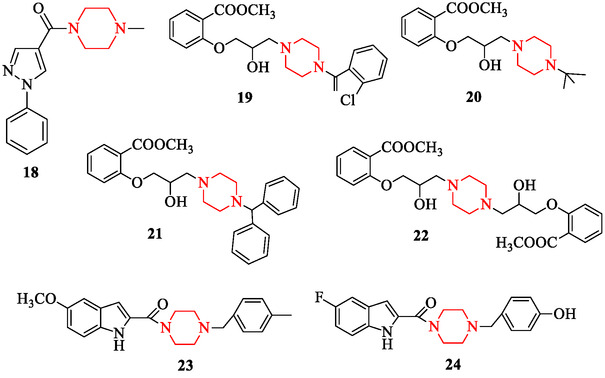
Anti‐inflammatory piperazine derivatives.

In vivo pharmacological validation is provided by Batista et al. [136], who synthesized (4‐methylpiperazin‐1‐yl)(1‐phenyl‐1H‐pyrazol‐4‐yl)methanone (compound 14) and demonstrated its capacity to suppress TNF‐α production in a murine model, without detectable acute oral toxicity (Figure 11, compound 18).

Yu et al. [137] reported that naphthaleneoxypropargyl‐containing piperazine derivatives efficiently modulate immune effector cell populations in aseptic inflammation models, confirming their immunoregulatory potential and expanding the anti‐inflammatory relevance of this chemical class. Li et al. [138] extended this class by preparing a series of methyl salicylate derivatives bearing a piperazine moiety (compounds 19–22). These analogs were evaluated in xylene‐induced ear edema and carrageenan‐induced paw edema in mice; at 100 mg/kg, many derivatives matched or exceeded the anti‐inflammatory effect of aspirin (100 mg/kg), with compound 22, dimethyl 2,2′‐((piperazine‐1,4‐diylbis(2‐hydroxypropane‐3,1‐diyl))bis(oxy))dibenzoate, showing the greatest potency (Figure 11, compounds 19–22). The hybridization strategy has also yielded promising dual‐action molecules. Baydar et al. [139] reported 5‐substituted indole derivatives with a piperazine subunit (compounds 23, 24), which showed antioxidant and anti‐inflammatory activity (with IC_50_ = 0.68 mM and 0.33 mM, respectively), outperforming unsubstituted analogs. Their docking analyses suggest good binding affinity to inflammatory enzyme targets (Figure 11, compounds 23, 24).

Supporting recent findings, Martins et al. [140] studied another novel piperazine‐based derivative, LQFM202, and demonstrated its antinociceptive and anti‐inflammatory effects in vivo, further reinforcing the translational potential of piperazine scaffolds in inflammation models. Moreover, the broader bioactivity review of piperazine derivatives [33, 141] provides an overview of both classical and novel anti‐inflammatory applications, including mechanistic insights and SAR trends [142].

Similarly, Balabekova et al. [143] investigated the immunomodulatory action of a fluorinated piperazine derivative MXF‐19 under metal‐induced immunodepression in rats and observed restoration of T‐regulatory lymphocyte populations and attenuation of aseptic inflammation, further validating the role of piperazine scaffolds in immune homeostasis and anti‐inflammatory responses.

In summary, the examples above illustrate how aromatic or heterocyclic substitution onto piperazine cores can significantly enhance anti‐inflammatory efficacy. Given the current interest in targeting cytokine storms or hyperinflammatory states (e.g., in viral pneumonia or autoimmune exacerbations), future studies should evaluate these scaffolds in LPS‐induced systemic inflammation, cytokine profiling, and in silico docking/MD to COX, LOX, and cytokine receptor enzymes.

### 4.5 Antitubercular Activity

**FIGURE 12:**
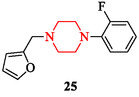
Chemical structure of 1‐(2‐fluorophenyl)‐4‐(furan‐2‐ylmethyl)piperazine.

Tuberculosis (TB), caused by *Mycobacterium tuberculosis*, remains a leading cause of mortality worldwide, with 1.5 million deaths annually reported by the World Health Organization. The continuous emergence of multidrug‐resistant (MDR) strains necessitates the search for novel antitubercular agents with new structural classes and mechanisms of action. Piperazine scaffolds received significant interest in this field due to their synthetic accessibility, favorable pharmacokinetics, and ability to host diverse substituents that modulate bioactivity [144]. Researchers in Ethiopia synthesized a series of novel 1,4‐disubstituted piperazine derivatives incorporating indole, furan, and pyridine motifs, each with established bioactivity profiles [145]. Among these, 1‐(2‐fluorophenyl)‐4‐(furan‐2‐ylmethyl)piperazine (compound 25) demonstrated pronounced inhibition against the *M. tuberculosis* H37Rv strain (Figure 12). This highlights the contribution of fluorine substitution and heteroaryl linkers in enhancing antimycobacterial potency.

**FIGURE 13:**
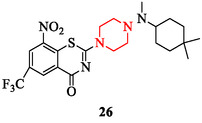
Chemical structure of 2‐(4‐((4,4‐dimethylcyclohexyl)(methyl)amino)piperazin‐1‐yl)‐8‐nitro‐6‐(trifluoromethyl)‐4H‐benzo[e][1,3] thiazin‐4‐one.

In another study, *N*‐(amino)piperazinylbenzothiazinones were developed as candidate agents against drug‐sensitive (*M. tuberculosis* H37Rv) and MDR (MDR‐MTB) strains [146]. The lead molecule, 2‐(4‐((4,4‐dimethylcyclohexyl)(methyl)amino)piperazin‐1‐yl)‐8‐nitro‐6‐(trifluoromethyl)‐4H‐benzo[e][1,3]thiazin‐4‐one (compound 26), exhibited strong inhibitory activity, low toxicity in rats (LD_50_ > 500 mg/kg), minimal cardiotoxicity, and favorable oral bioavailability (Figure 13).

SAR observations confirm that electron‐withdrawing groups such as fluorine, trifluoromethyl, and nitro substituents increase antimycobacterial activity, while heteroaryl substituents (furan, pyridine) improve lipophilicity and target engagement. Bulky aliphatic side chains at the piperazine nitrogen contribute to lower toxicity and better pharmacological profiles [145, 146]. Recent reviews support these findings, emphasizing the piperazine scaffold as a versatile template for antimycobacterial optimization [4, 146].

These results indicate that piperazine‐based derivatives are promising antitubercular candidates, particularly for drug‐resistant TB. Future studies should address metabolic stability, macrophage penetration, and intracellular accumulation to advance these molecules toward clinical application [4].

### 4.6 Antitumor Activity

**FIGURE 14:**
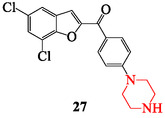
Structure of (5,7‐dichlorobenzofuran‐2‐yl)(4‐(piperazin‐1‐yl)phenyl)methanone.

Piperazine derivatives have been extensively studied as anticancer candidates due to their ability to interact with diverse molecular targets and modulate apoptosis, proliferation, and cell cycle pathways. Their synthetic flexibility supports the design of hybrid structures with enhanced cytotoxicity against solid and hematological malignancies [125, 147]. Several piperazine‐based compounds have shown activity in preclinical and early clinical evaluations, particularly against breast, colon, lung, prostate, and leukemia cell lines [31]. One study reported benzofuran‐piperazine hybrids as promising antitumor agents. The lead compound, (5,7‐dichlorobenzofuran‐2‐yl)(4‐(piperazin‐1‐yl)phenyl)methanone (compound 27), showed strong inhibition of proliferation across six cancer cell lines. Mechanistic evaluation suggested necrosis as the main pathway of cell death, and in vivo studies confirmed tolerability and tumor suppression in rats (Figure 14) [32]. Another important example is the synthesis of 4‐(*N*‐dithiobenzylpiperazine)‐1,8‐naphthalimide derivatives, which exhibited potent anticancer activity with improved safety profiles [148].

Several antitumor investigations rely only on cell‐viability endpoints without confirming pathway selective events. Assay panels often include only one or two cell lines, which restricts broader interpretation. Reports with high potency values rarely include information on stability, solubility, or metabolic characteristics, which introduces uncertainty regarding translational relevance. These gaps introduce uncertainty in the reported value of several antitumor candidates.

SAR analysis reveals that electron‐withdrawing substituents such as chloro, nitro, and trifluoromethyl groups enhance cytotoxic potency by stabilizing drug–target interactions, while benzofuran and naphthalimide linkers increase lipophilicity and facilitate DNA intercalation or topoisomerase inhibition. Substitution with dithiobenzyl groups at the piperazine nitrogen improves tumor selectivity and decreases off‐target toxicity, and bulky aromatic groups at the N‐4 position contribute to cell cycle arrest and apoptosis induction [32, 149]. Recent findings further expand these principles. Schumacher et al. [32] confirmed the efficacy of benzofuran‐piperazine analogs with selective necrotic mechanisms, while Liang et al. [148] reported 4‐(*N*‐dithiobenzyl)piperazine‐naphthalimide conjugates as multitarget antitumor agents with low resistance risk. Moreover, novel piperazine‐based delivery systems, including siRNA‐conjugated constructs, are being explored to enhance tumor selectivity and minimize systemic toxicity.

Despite the range of biological activity demonstrated by piperazine derivatives, their translation from preclinical discovery to clinical approval has remained limited. Many candidates show pharmacokinetic drawbacks such as low oral bioavailability, rapid metabolic clearance, and insufficient penetration across the blood‐brain barrier, which restrict their therapeutic applicability [105, 149]. Off‐target interactions and scaffold‐related toxicities also complicate development, as the dual‐nitrogen core, while conferring versatility, increases the likelihood of polypharmacology. In the antimicrobial field, rapid resistance development diminishes the long‐term value of promising leads, and in oncology similar issues of poor stability and biodistribution hinder progress. Computational methods such as docking and QSAR frequently predict strong binding affinities, yet these outcomes rarely translate into reliable in vivo efficacy because of oversimplified models that fail to capture biological complexity. Regulatory advancement is often delayed by incomplete ADME and toxicology validation, contributing to attrition during clinical trials. Addressing these challenges calls for continued optimization of substituents, hybridization strategies, and delivery systems, supported by integrated design pipelines that combine computational predictions with rigorous pharmacological evaluation and translational modeling [105, 149].

## 5 Conclusion

This review summarizes recent progress in the chemistry and pharmacology of piperazine derivatives. Synthetic advances include *N*‐substitution, reductive amination, cross‐coupling, C—H activation, microwave‐assisted routes, and flow‐based protocols. These strategies expand access to diverse substitution patterns suited for medicinal design. Structure–activity data show that aryl, heterocyclic, and hybrid groups increase target affinity and improve pharmacokinetic behavior. Antimicrobial, anticancer, and neuroactive profiles remain prominent, yet multiple series show limited stability and incomplete translational assessment. Reported activity claims often lack solubility data, metabolic information, or selectivity profiling, which restricts interpretation. Several computational reports present predicted affinities without biological validation, which lowers confidence in reported performance. Stronger links between synthesis, mechanistic evaluation, and experimental pharmacology are needed to establish reliable SARs. Integration of predictive modeling and cheminformatics tools will support precise lead refinement. These directions provide a path toward piperazine‐based agents with improved efficacy and safety.

## Author Contributions

**Assel Ten**: conceptualization (equal), project administration (lead), supervision (equal), validation (equal), visualization (equal), writing – original draft (equal), writing – review & editing (equal). **Raushan Koizhaiganova**: conceptualization (equal), methodology (equal), supervision (equal), visualization (equal), writing – original draft (equal), writing – review & editing (equal). **Dilnaz Bissenbay**: formal analysis (supporting), resources (supporting). **Bagila Tursynova**: formal analysis (supporting), resources (supporting), software (supporting). **Zhanar Zhaxibayeva**: conceptualization (equal), resources (supporting), visualization (equal), writing – original draft (equal). **Valentina Yu**: conceptualization (lead), project administration (lead), supervision (lead).

## Funding

This study was supported by Science Committee of the Ministry of Science and Higher Education of the Republic of Kazakhstan (AP23484420, BR21882220).

## Conflicts of Interest

The authors declare no conflicts of interest.
